# A Microfluidic Chip-Based MRS Immunosensor for Biomarker Detection *via* Enzyme-Mediated Nanoparticle Assembly

**DOI:** 10.3389/fchem.2021.688442

**Published:** 2021-05-28

**Authors:** Binfeng Yin, Changcheng Qian, Songbai Wang, Xinhua Wan, Teng Zhou

**Affiliations:** ^1^ School of Mechanical Engineering, Yangzhou University, Yangzhou, China; ^2^ School of Chemistry and Chemical Engineering, Shanxi University, Taiyuan, China; ^3^ Mechanical and Electrical Engineering College, Hainan University, Haikou, China

**Keywords:** magnetic relaxation switching, microfluidic chip, alpha-fetoprotein, immunosensor, nanoprobe

## Abstract

Conventional immunoassay methods have their common defects, such as tedious processing steps and inadequate sensitivity, in detecting whole blood. To overcome the above problems, we report a microfluidic chip–based magnetic relaxation switching (MRS) immunosensor *via* enzyme-mediated nanoparticles to simplify operation and amplify the signal in detecting whole blood samples. In the silver mirror reaction with catalase (CAT) as the catalyst, H_2_O_2_ can effectively control the production of Ag NPs. The amount of Ag NPs formed further affects the degree of aggregation of magnetic nanoparticles (MNP_S_), which gives rise to the changes of transverse relaxation time (T_2_). Both sample addition and reagent reaction are carried out in the microfluidic chip, thereby saving time and reagent consumption. We also successfully apply the sensor to detect alpha-fetoprotein (AFP) in real samples with a satisfied limit of detection (LOD = 0.56 ng/ml), which is superior to the conventional ELISA.

## Introduction

Immunoassays have always been irreplaceable methods in disease diagnosis, environmental monitoring, and medical treatment evaluation (Kingsmore, 2006; Rusling et al., 2010; Barbosa and Reis, 2017). Many methods, including gold immunochromatography assay (GICA) (Wang et al., 2018; Yang et al., 2021), chemiluminescent immunoassay (CLIA) (Iranifam, 2014; Fan et al., 2021), and enzyme-linked immunoassay (ELISA) (Raleigh et al., 1992; Zhang et al., 2019), were designed for tumor marker detection. The above conventional methods have their superiority and have been applied to clinical practice. However, they also have their intrinsic shortcomings. GICA is always accompanied by the problems such as low sensitivity, false positives, and inaccurate quantification due to simple operation and imprecise testing equipment (Giljohann et al., 2010; Ji et al., 2019). CLIA is a highly sensitive detection method, but it uses chemiluminescent substances as markers, which leads to defects such as high background values, poor stability, and low reproducibility (Zhang et al., 2018; Xiao and Xu, 2020). As a typical commercialized product, ELISA has the advantage of high stability and good repeatability, while elaborate preprocessing and washing steps lead to the strict requirement for professional operators (Zhao et al., 2019; Sheng et al., 2021). Therefore, there is an urgent need for a low background, high-throughput, and easy-to-operate system to achieve rapid, sensitive, and accurate analysis of targets.

Recently, people have devoted much energy to the exploitation of novel biosensors for detecting biomarkers (Jia et al., 2017; Cui et al., 2020; Huang et al., 2020). Magnetic relaxation switching (MRS) sensors based on nuclear magnetic resonance (NMR) are becoming widespread focus (Choi et al., 2017; Tian et al., 2017; Chen et al., 2018b; Dong et al., 2018) in biochemical analysis for their low background, high sensitivity, rapidness, and simplicity. MRS assays have been employed as a powerful tool to identify and quantify a wide range of targets, including proteins (Liang et al., 2011), microorganisms (Zhao et al., 2019), nucleic acids (Liong et al., 2013), small molecules (Atanasijevic et al., 2006), and other biomarkers. This method changes the T_2_ value of neighboring water protons of magnetic nanoparticles along with their state switching between dispersion and aggregation. And the changes of ΔT_2_ correlate with the concentration of the target in the sample. Thus, ΔT_2_ can be used as analytical signal in MRS immunosensor. Usually, negligible magnetic substance existed in biological samples. Compared with electrochemical (Wang D. et al., 2015; Fan et al., 2020; Song et al., 2020) or optical (Walling et al., 2009; Zhou et al., 2015; Yin et al., 2017) sensors, it has the advantages of lower background value. Thus, it can be used to detect opaque samples, such as muddy water (Jia et al., 2017; Porion and Delville, 2020), milk (Chen et al., 2013; Wang S. et al., 2015), and whole blood (Neely et al., 2013; Mylonakis et al., 2015) directly. However, the MRS sensor suffers from its relatively low sensitivity and not-so-simple preprocessing steps when it comes to the analysis of trace substance in complex samples (Yin et al., 2016). According to a report, one kind of magnetic/silver nanoassemblies (Ag-MNPs) mediated by an enzyme cascade reaction can greatly enhance the state change of MNPs (from dispersed state to aggregated state). Therefore, it can improve the sensitivity of the conventional MRS sensor dramatically (Chen et al., 2018a).

Microfluidic chips have a good prospect in immunoassay because they can be integrated with different elements such as pumps, valves, and electronics (Chao et al., 2016; Wang and Fu, 2018; Wolf et al., 2018). Reagent storage, protein adsorption, sample separation, and microfluid flow characteristics and direction control in microfluidic chips have become the off-the-shelf technique. Thus, microfluidic chips have the potential to replace tedious pretreatment procedures (Rothbauer et al., 2018).

**FIGURE 1:**
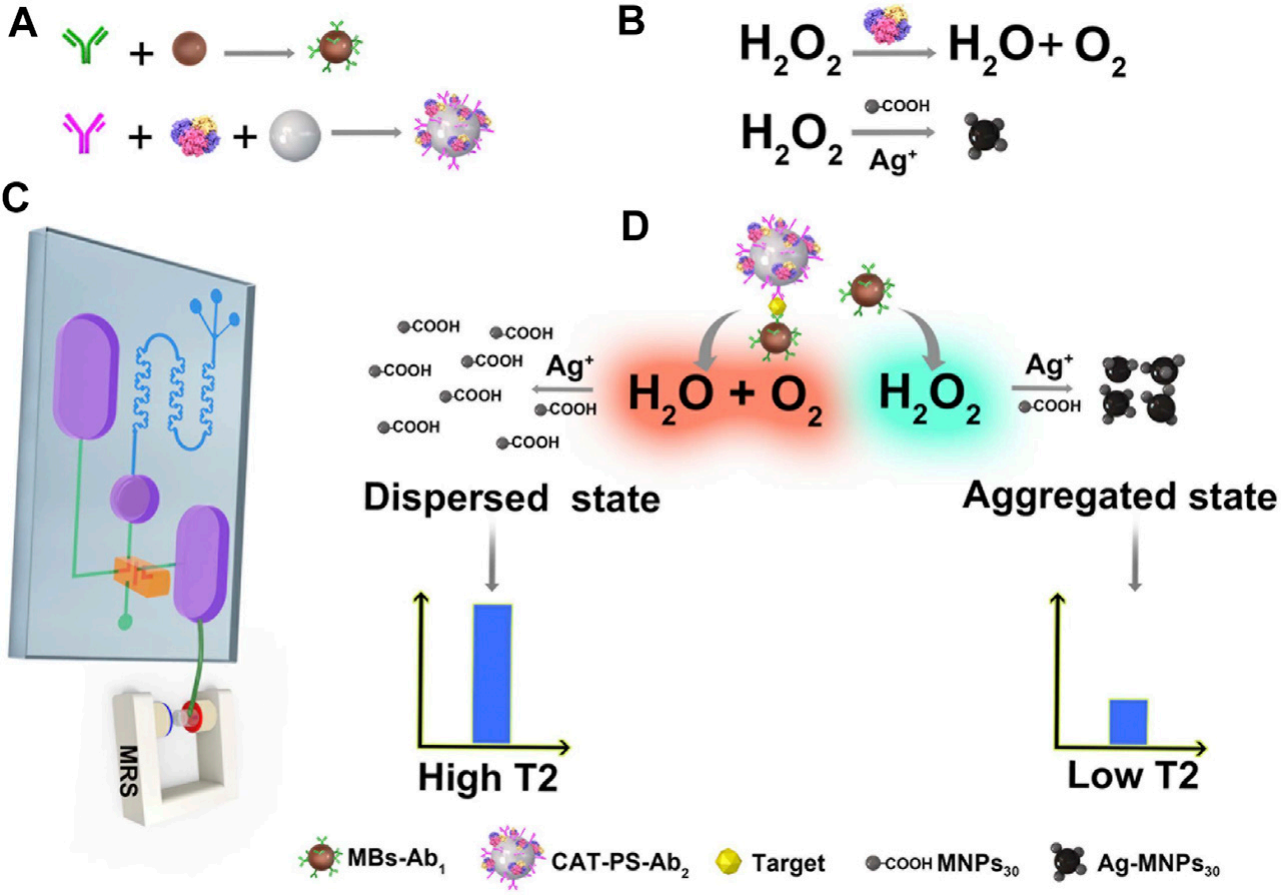
**(A)** Generation of MBs labeled capture antibody (MBs-Ab1) and PS microparticle labeled both CAT and detection antibody (CAT-PS-Ab2). **(B)** Role of CAT in the formation of Ag-MNPs. **(C)** A microfluidic chip–based MRS immunosensor for biomarker detection. **(D)** CAT labeled on the surface of PS can catalyze H_2_O_2_ and mediate the assembly aggregation of Ag-MNPs, which results in a lower NMR signal.

To realize low cost, high sensitivity, and rapid detection for the tumor biomarkers such as alpha-fetoprotein (AFP), herein, we developed an amplified MRS immunosensor *via* enzyme-mediated cascade reaction with a microfluidic chip. First, we labeled the capture antibody (Ab_1_) on magnetic beads (MBs) to generate MB-Ab_1_. We labeled the enzyme catalase (CAT) and detection antibody (Ab_2_) on polystyrene (PS) microspheres at the same time to prepare CAT-PS-Ab_2_ for forming an enzymatic amplification system (Figure 1A). Then the two conjugations (MBs-Ab_1_ and CAT-PS-Ab_2_) and analyte are injected into the snake-shaped channel of the microfluidic chip and are mixed *via* fluid inertia. The targets (T) can specifically bind antibodies modified on MBs and PS to form a sandwich structure (MBs-Ab_1_-T-Ab_2_-PS-CAT). An NdFeB magnet is placed at the bottom of the storage chamber to enrich the MBs. The pressure valve on the right controls the direction of the fluid, and realizes the functions of washing and reagent-adding (Figure 1C). CAT possesses a high catalytic effect on the substrate H_2_O_2_ to generate water and oxygen, which can adjust the aggregated degree of Ag-MNPs through the decomposition of H_2_O_2_ (Figure 1B). When 30 nm carboxyl-modified magnetic nanoparticles (MNPs_30_-COOH), Ag^+^, and H_2_O_2_ are mixed without CAT, Ag^+^ is reduced by sufficient H_2_O_2_ to silver nanoparticles (Ag NPs) immediately, and fewer Ag^+^ was adsorbed or diffused on the surface of magnetic nanoparticles, which hindered the formation of Ag-MNPs_30_. While there is CAT existing, the concentration of H_2_O_2_ is controlled at a relative lower level, and the formation of Ag NPs is retarded. Abundant Ag^+^ on the surface of magnetic particles can facilitate the aggregation of Ag-MNPs_30_ (Figure 1D). Finally, the NMR signal of Ag-MNPs_30_ was recorded.

## Experimental Section

### Materials and Apparatus

Alpha-fetoprotein (AFP, 1 mg/ml), anti-AFP capture antibody (Ab_1_, 5 mg/ml), anti-AFP detection antibody (Ab_2_, 5 mg/ml), carcinoembryonic antigen (CEA, 1 mg/ml), and human IgG (IgG, 10 mg/ml) were obtained from Hotgen Biotech Inc. (Beijing, China). Catalase (CAT) is from bovine liver, 1-ethyl-3-[3-dimethylaminopropyl] carbodiimide hydrochloride (EDC), N-hydroxysulfosuccinimide (sulfo-NHS), and bovine serum albumin (BSA) were purchased from Sigma-Aldrich (Shanghai, China). 30 nm carboxylic acid–modified magnetic nanoparticles (5 mg/ml) and 30 nm amino-modified magnetic nanoparticles (5 mg/ml) were purchased from Ocean NanoTech, LLC (United States), 250 nm carboxylic acid–modified magnetic beads (MBs, 10 mg/ml) were purchased from micromod Partikeltechnologie GmbH (Germany), and carboxyl-functionalized polystyrene microspheres (PS, 100 mg/ml, d = 1 µm) were purchased from the Bangs Laboratories, Inc. (United States). Hydrogen peroxide (H_2_O_2_) was obtained from Beijing Chemical Works (China). Potassium hydroxide (KOH), AgNO_3_, and NH_3_·H_2_O were obtained from Beijing Chemical Reagents Co., Ltd (China). Polydimethylsiloxane (PDMS) and curing agent (Sylgard 184) were purchased from Dow Corning Inc. (MI, United States). Tablets of phosphate buffer saline (PBS, 0.01 M, pH 7.4) and Tween-20 were purchased from Amresco LLC (United States). Milli-Q water was generated throughout a Millipore water system (United States).

The PT-10s plasma cleaner for PDMS bonding was produced by Shenzhen Sanhoptt Co. Ltd (China). 1.5T (60 MHz) nuclear magnetic resonance (NMR) spectrometer system for the measurement of transverse relaxation time was obtained from Shanghai Huantong Science and Education Equipment Co., Ltd (China). JEM-2100F (200 kV) field emission transmission electron microscope (TEM) for characterization of nanoparticle morphologies was obtained from Japan Electron Optics Laboratory Ltd (Japan). 2,300–001M EnSpire multimode plate reader was obtained from PerkinElmer Inc (United States). Zetasizer Nano ZS (Malvern Panalytical Ltd, United Kingdom) dynamic light scattering (DLS) was used for particle size and zeta potential measurements. The SLA 3D printer for manufacture of molds was provided by Yangzhou SHINING 3D Co. Ltd (China).

### Design of the Snake-Shaped Microfluidic Chip

**FIGURE 2:**
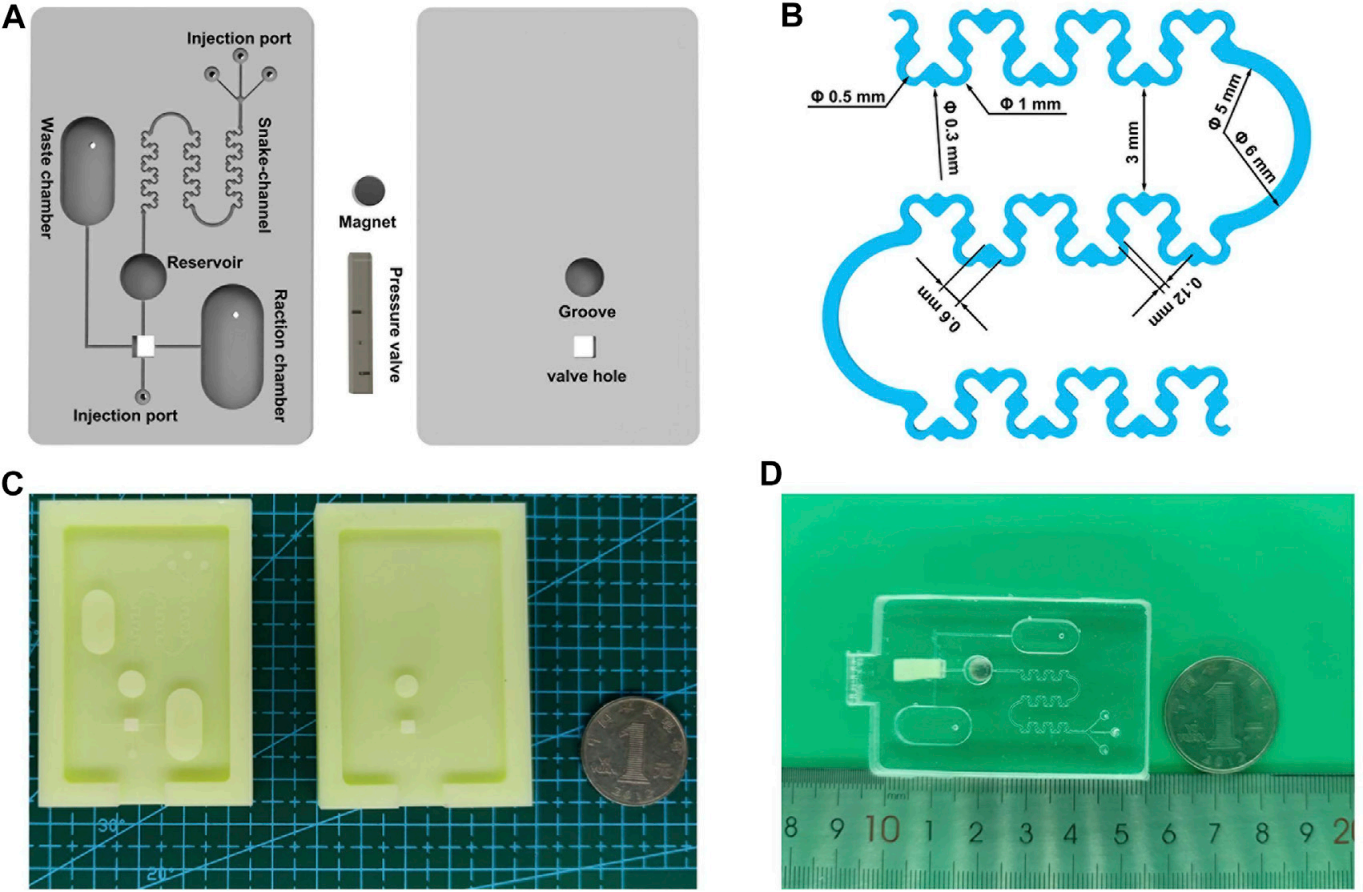
**(A)** Schematic diagram of SSMC. **(B)** Optimized size of snake-shaped mixing channel. **(C)** The molds of microfluidic chips. **(D)** A picture of real SSMC.

The components and cast molds of snake-shaped microfluidic chip (SSMC) are designed using Unigraphics NX 10.0 3D modeling software (Siemens PLM Software, Berlin, Germany). Specifically, the components of the SSMC consist of three parts: PDMS channel layer, PDMS substrate layer, and NdFeB magnet, and three pressure valve stages (Figure 2A and Figure S1). The PDMS channel layer (35 mm* 55 mm* 3.5 mm) has a total of four injection ports. The three injection ports on the top converge into the snake-shaped channel in 45° interval arrangement. We have optimized the design of the snake-shaped channel to mix the sample sufficiently. To be specific, three narrow-broad repeated units were used. The diameter of the spacious and thin channels is 0.6 mm and 0.12 mm, respectively. To make the sample achieve efficient mixing, and uneven flow velocity and pressure in the channel (Figure 2B), there is a valve hole (2.8 mm* 2.8 mm) for installing pressure valve on the lower side. It is connected to the waste chamber (200 μl), the bottom injection port, the reaction chamber (280 μl), and the reservoir (50 μl). The PDMS substrate layer (35 mm* 55 mm* 3 mm) is dug into a circular groove to place the magnet. There is also a valve hole corresponding to the PDMS channel layer below it. The pressure valve (3 mm* 3 mm) and the valve hole are assembled by an interference fit. It is designed with three holes (0.6 mm* 0.6 mm) in height to control the flow direction in the channel. An NdFeB magnet is fixed in the groove of the substrate layer.

### Fabrication and Assembly of the SSMC

First, we designed the three-dimensional model of molds and pressure valve by the 3D modeling software. Then we used the SLA 3D printer to automatically print the molds and pressure valve with the photosensitive resin (Figure 2C). After printing, the mold is cleaned in an ultrasonic cleaning machine containing alcohol to remove unmolded resin on the surface. A 9.5:1 (w/w) mixture of PDMS matrix and curing agent was vacuumized in the vacuum drying oven. The dual PDMS layer molds including uniform and bubble-free mixture were placed in a thermostatic oven at 75°C for 1.5 h, and then the dual PDMS layer in the mold was removed with tweezers. The next step is to assemble the SSMC chip as follows: 1) disposing the dual PDMS layers in the plasma cleaner for 60 s (220 w working power and 2.0 l/min gas flow rate), 2) slowly squeezing out the bubbles of the chip by hand and make them stick together, and 3) fixing the pressure valve on the dual PDMS layers to ensure the first hole aligned with the chip channel (Figure 2D).

### Preparation of Ag(NH_3_)_2_OH Solution

To prepare the Ag(NH_3_)_2_OH solution (Tollen’s reagent), 200 µl of NH_3_·H_2_O (15 M) was dropwise added into 6 ml of AgNO_3_ (0.1 M), while stirring mixture solution until the brown precipitate dissolved. Then 3 ml of KOH solution (0.8 M) was added, and the brown precipitate reformed. To dissolve the precipitate, 200 µl of NH_3_·H_2_O was added again. Finally, deionized water (Milli-Q water) was added to a final volume of 25 ml, and was stored in the dark at 4°C.

### Preparation of MB-Ab_1_ Conjugate

First, 5 mg of suspended MBs was transferred into 1 ml of the MES buffer (80 nM, pH 6.0). After that, 80 µl of EDC (10 mg/ml) and 40 µl of NHS (10 mg/ml) were added, and the mixture was activated for 0.5 h at room temperature (RT). The activated MBs were washed three times using 1 ml of PBS buffer (pH 7.4, 0.01 M) with a magnetic separator, and then were dispersed in 0.5 ml of PBS solution. 0.5 mg of Ab1 was added into the above solution and stirred with 300 rpm for 1.5 h at RT. After that, 0.5 ml of 3% BSA solution was added to block the surface of MBs for 0.5 h. The MB-Ab1 conjugate was magnetically separated from the free Ab1, and the conjugate was washed three times using 1 ml of PBST buffer (PBS buffer with 0.5% Tween-20). The MB-Ab_1_ conjugate was resuspended in 0.5 ml of PBS with 0.1% BSA and stored at 4°C for further use.

### Preparation of CAT-PS-Ab_2_ Conjugate

First, 10 mg of suspended PS microspheres was transferred into 0.9 ml of deionized water in a clean ultrafiltration tube (100 kDa filter), and then was centrifuged at 8,500 rpm for 10 min. The collected PS microspheres were resuspended in 0.5 ml of deionized water, and 120 µl of EDC (10 mg/ml) and 60 µl of NHS (10 mg/ml) were added to it. The reaction mixture was stirred with 300 rpm for 30 min at RT, and was diluted using 1 ml of PBS buffer (pH 7.4, 0.01 M). After that, different quantities of Ab_2_ and CAT were added into the microparticle suspension. The above mixture solution was stirred mildly for 2 h at RT. Then 200 µl of 5% BSA solution was subsequently added and wobbled gently for 30 min. The obtained mixture was centrifuged for 10 min at 6,000 rpm, and was resuspended in 1 ml of PBS solution. The previously mentioned centrifuged and resuspended steps were repeated three times. The CAT-PS-Ab_2_ conjugate was resuspended in 1 ml of PBS (pH 7.4, 0.1% BSA) and stored at 4°C for further use.

### Procedures of Detecting AFP and Samples

**FIGURE 3:**
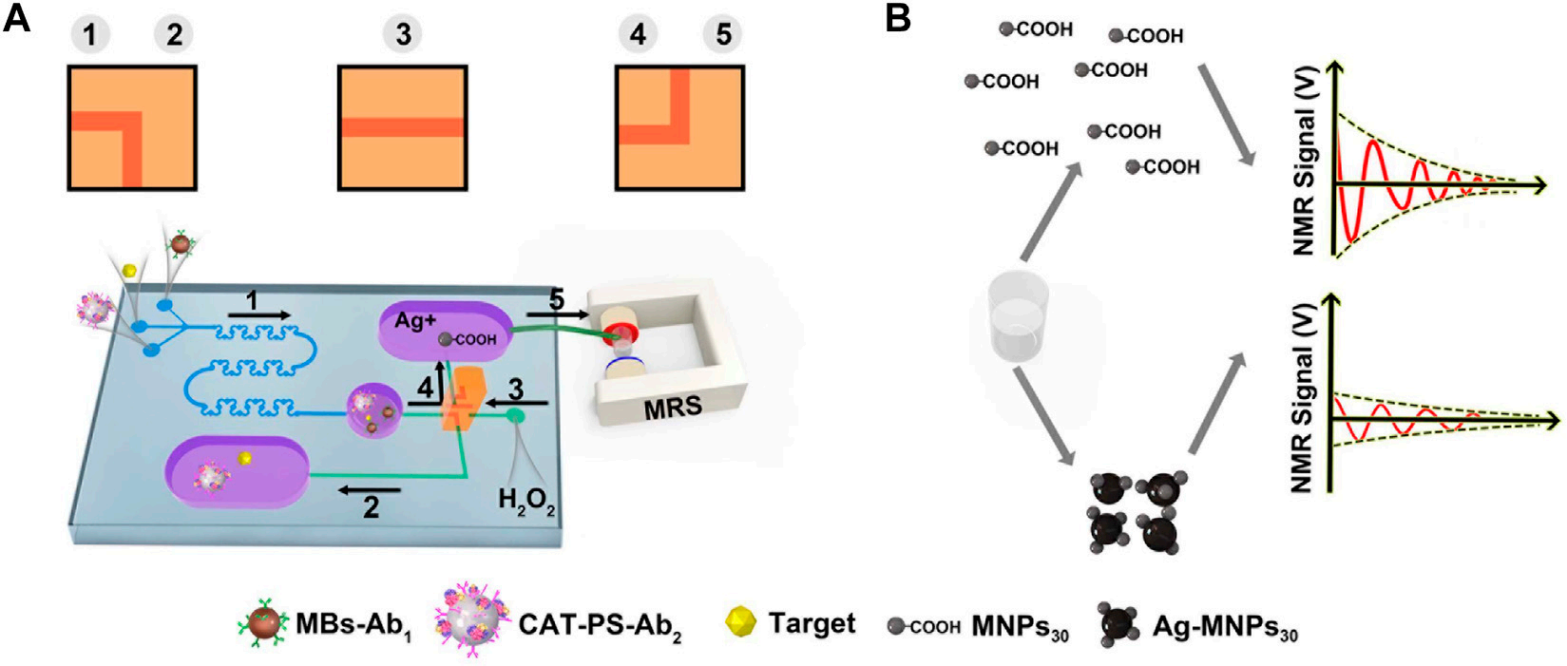
**(A)** The process of detecting AFP and the direction of the pressure valve at each step. **(B)** Different NMR signals in the dispersed state and the aggregated state.

First, we add 160 μl Tollen’s reagent (2.4 mM) and 20 μl MNPs_30_-COOH (0.5 μg/ml) into the reaction chamber of SSMC. Then the detecting procedures are carried out as follows: 1) we add 20 µl of MB-Ab1 (0.1 mg/ml), 100 µl of different concentrations of AFP (1,280 ng/ml, 640 ng/ml, 320 ng/ml, 160 ng/ml, 80 ng/ml, 40 ng/ml, 20 ng/ml, 10 ng/ml, 5 ng/ml or 2.5 ng/ml, and 0.625 ng/ml or 0 ng/ml), and 20 µl of CAT-PS-Ab_2_ (0.05 mg/ml) in the injection ports separately. The magnet absorbs the MB-Ab1-T-Ab2-PS-CAT conjugates mixed by snake-shaped channel, and uncombined MB-Ab_1_ are discarded in the reservoir. 2) After 15 min, we wash the conjugates remaining in the channel with PBS (0.01 M, pH 7.4), and uncombined AFP and CAT-PS-Ab_2_ are washed into the waste chamber. 3) Press the pressure valve to ensure the second hole aligned with the chip channel. We add 20 µl H_2_O_2_ (250 µM) from bottom injection port to the reservoir. The conjugates react with H_2_O_2_ for 5 min. 4) Open the reaction chamber through the third hole aligned with channel. The suspension in the reservoir is flowed into the reaction chamber (Figure 3A). 5) After 5 min, we use the NMR analyzer to measure the ΔT_2_ value of 20 µl extracted mixture from reservoir (Supplementary Figure S2).

The parameters of the inversion recovery pulse sequences for ΔT_2_ measurements are as follows: NMR frequency, 62.16 MHz; pulse separation, 10 m; 90° pulse width, 32 μs; 180° pulse width, 64 μs; number of scans, one; and repetition time, 10 s. To obtain the limit of detection (LOD), we employ the following formula: LOD = 3S/M (where S is the value of the standard deviation of blank samples and M is the slope of standard curve within the low-concentration range).

When MNPs are in a dispersed state, they show a higher NMR signal. Once Ag-MNPs30 probe was formed, it shows a lower NMR signal due to the aggregated state of MNPs (Figure 3B). To control the flow direction of the reagent in the channel at each step, the pressure valve is switched as shown in Figure 3A.

## Results and Discussion

### Surface Charge of MNPs/Ag-MNPs_30_

**FIGURE 4:**
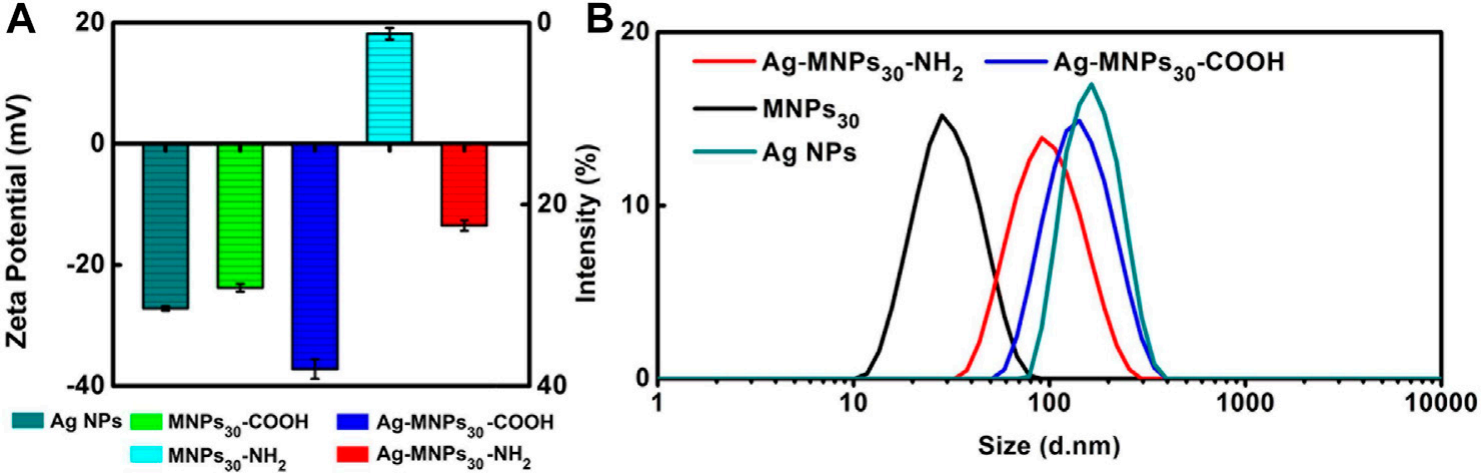
**(A)** Zeta potentials of Ag NPs, MNPs_30_-COOH, Ag-MNPs_30_-COOH, MNPs_30_-NH2, and Ag-MNPs_30_-NH2. **(B)** DLS analysis of Ag NPs, MNPs_30_-COOH, Ag-MNPs_30_-COOH, MNPs_30_-NH2, and Ag-MNPs_30_-NH2.

The difference in surface charge of MNPs_30_ significantly affects the formation of Ag NPs. We tested the Zeta potentials of MNPs_30_-COOH, Ag-MNPs_30_-COOH, MNPs_30_-NH_2_, and Ag-MNPs_30_-NH_2_ (Figure 4A). Results indicate that only MNPs_30_-NH_2_ have a positively charged surface (ζ = 26 mV), and MNPs_30_-COOH show a negatively charged surface (ζ = −13.5 mV). Whether positively charged or negatively charged MNPs can combine with negatively charged Ag NPs (ζ = −27.2 mV), charges in the surface of MNPs will become more negative. It means that Ag NPs in quantity gathered on the surface of magnetic nanoparticles.

DLS results show that the average sizes of both Ag-MNPs_30_-NH_2_ (105 nm) and Ag-MNPs_30_-COOH (135 nm) are larger than those of magnetic particles itself, that is, MNPs_30_ (30 nm) (Figure 4B), which indicates that both MNPs_30_-COOH and MNPs_30_-NH_2_ can be aggregated with Ag NPs. It is consistent with the result of zeta potential results.

### Formation and Characterization of Ag-MNPs_30_

**FIGURE 5:**
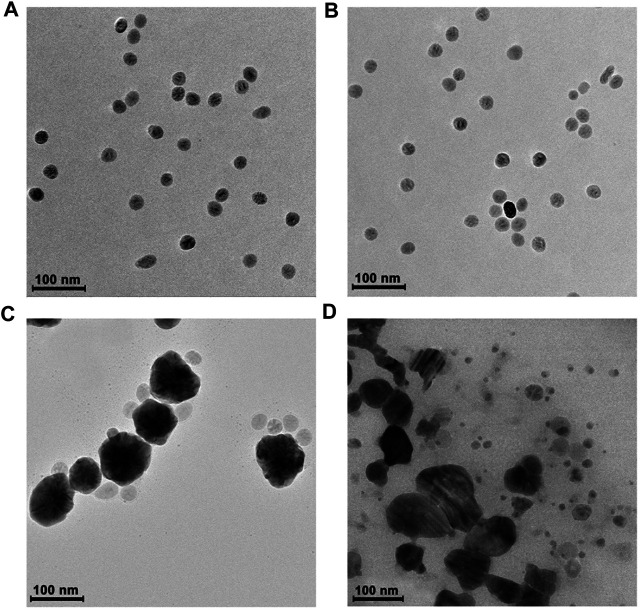
Characterization of MNPs_30_-COOH, MNPs_30_-NH_2_, Ag NPs, Ag-MNPs_30_-COOH, and Ag-MNPs_30_-NH_2_ by transmission electron microscope (TEM). **(A)** The shape of MNPs_30_-COOH. **(B)** The shape of MNP_30_-NH_2_. **(C)** The appearance of Ag-MNPs_30_-COOH. **(D)** The appearance of Ag-MNPs_30_-NH_2_.

We selected one positively charged surface (MNPs_30_-NH_2_) and one negatively charged surface (MNPs_30_-COOH) of magnetic nanoparticles to prepare the magnetic/silver nanoassemblies to select the appropriate magnetic nanoparticles to carry out the next experiment. We utilize TEM to observe the morphology and structure of Ag-MNPs_30_-COOH and Ag-MNPs_30_-NH_2_. When the concentrations of MNPs_30_-COOH and MNPs_30_-NH_2_ are at 5 μg/ml, the shapes and diameters of the two particles are almost the same (Figures 5A,B). Once Tollen’s reagent and H_2_O_2_ are pouring into the magnetic nanoparticle solution, the results showed an obvious distinction. In MNPs_30_-COOH solution, a well-arranged flower-like structure attributed to Ag-MNPs_30_-COOH was founded (Figure 5C). However, a blended and irregular structure of Ag-MNPs_30_-NH_2_ was observed when MNPs_30_-NH_2_ was used as the substrate in the same condition. Most of MNPs_30_-NH_2_ are still dispersed in the solution and do not absorb on the surface of Ag NPs (Figure 5D).

The reasons why MNPs with negative charged surface can form the regular shape are as follows (Wang et al., 2009; Gilroy et al., 2016): First, Ag^+^ is adsorbed on the negatively charged surface of MNPs_30_, and a local concentration difference of positive charge was formed. Then, the enriched Ag^+^ is reduced by H_2_O_2_ to Ag NPs and finally forms the uniformed Ag-MNPs probe.

For comparison, we also photographed the morphology of pure Ag NPs without MNPs_30_-COOH (Supplementary Figure S3). It showed that the shape of Ag NPs was not uniform. The surface atoms on the small size of Ag NPs have higher energy than the interior material. According to the principle of minimum energy, Ag NPs would spontaneously tend to aggregate to form irregular accumulation. The above results suggest that pure Ag NPs and Ag-MNPs_30_-NH_2_ cannot form a regular shape as required in our experiment, while MNPs_30_-COOH with negatively charged surface can get uniform size of Ag-MNPs_30_ for further use.

In addition, we employ UV-Vis spectrum to testify the principle of Ag NPs-MNPs_30_ assembly probe formation. Individual MNPs_30_-COOH or MNPs_30_-NH_2_ has no obvious characteristic absorption from 300 to 700 nm (Supplementary Figure S4). After injection of H_2_O_2_, the color of the Ag^+^ solution turns black, and a strong absorption peak appears at 450 nm, indicating that H_2_O_2_ can promote the formation of Ag NPs. The color of the solution gradually turns brown, and the absorption peak has a hypochromatic shift to 400 nm when MNPs_30_-COOH was used as the substrate, while the color only becomes weak, and absorption decreases with it when MNPs_30_-NH_2_ was used. These comparisons explain that negatively charged MNPs_30_-COOH interacts with Ag NPs to promote the formation of probe better than MNPs_30_-NH_2_. This is consistent with the above results.

### Response Performance of Ag-MNPs_30_ to H_2_O_2_

As one of the substrates in the redox system of Ag^+^ and H_2_O_2_, the amount of H_2_O_2_ consumed determined the amount of Ag NPs or Ag-MNPs_30_ generated. The Ag NPs can be used as optical signal and Ag-MNPs_30_ can be used as magnetic signal readout respectively. Hence, we investigate the response of Ag NPs or Ag-MNPs_30_ toward H_2_O_2_ by ultraviolet absorption spectrum and MRS.

First, we dilute MNPs_30_-COOH from 0 μg/ml to 50 μg/ml (Supplementary Figure S5). With the increase of concentration of MNPs, the ΔT_2_ value decreases as a ladder. That is a good proof that the concentration of MNPs_30_ has immediate effect on ΔT_2_. Next, we study the sensitivity and detection range of Ag-MNPs_30_ response to H_2_O_2_. When ΔT_2_ is employed for the readout, the detection range of 1.6 μM–10 mM was observed (Supplementary Figure S6A), and a linear relationship is 1.6 μM–1 mM ($y=26.5x+32.5,\;R^{2}=0.99,\;\mathrm{LOD}=0.53\;\mathrm{\mu M}$) (Supplementary Figure S6C). If there was an optical signal–based readout, the detection range for H_2_O_2_ would be 40 μM–10 mM (Supplementary Figure S6B), and a linear range 40 μM–5 mM was observed ($y=0.62x-0.21,\;R^{2}=0.94,\text{ LOD}=5.2\;\text{μM}$) (Supplementary Figure S6D). Therefore, compared with ultraviolet absorption detection, the MRS sensor has enhanced about 10 times sensitivity in the H_2_O_2_ catalytic reaction.

### Optimization of Experiment Condition of the MRS Sensor

The above result indicates that the ΔT_2_ generated by the MRS sensor is easily affected by the concentration of H_2_O_2_. The optimal ΔT_2_ value appeared at 1 mM H_2_O_2_. We need to further optimize the concentrations of Ag^+^ and MNPs_30_, reagent addition sequence, reaction time, and coupling ratio of CAT and Ab_2_ on the surface of the PS particles.

We added 0.05 μg/ml, 0.5 μg/ml, and 5 μg/ml MNPs_30_ to varying H_2_O_2_ concentrations. The gradient of ΔT_2_ at 0.5 μg/ml is the largest. By contrast, the changes in ΔT_2_ are the smallest when MNPs_30_ were added in 5 μg/ml (Supplementary Figure S7A).

Different orders of reagent addition would also affect the extent of reaction. We compared the results of two adding sequences: 1) MNPs_30_, Tollen’s reagent, and H_2_O_2_, and 2) Tollen’s reagent, H_2_O_2_, and MNPs_30_ (Supplementary Figure S7B). Obviously, the former is superior to the latter. It can be explained that H_2_O_2_ will reduce Tollen’s reagent first into Ag NPs and cannot form Ag-MNPs_30_ in the latter sequences. In order to simplify the chip design and operation, we first add H_2_O_2_, and then add the MNPs_30_ and Tollen’s reagent mixture. Under the above optimized conditions, we add different concentrations of Tollen’s reagent, and the maximum ΔT_2_ value is observed at 2.4 mM of Ag^+^ (Supplementary Figure S7C). And after 90 s, the reaction reached a plateau (Supplementary Figure S7D). Hence, the optimal concentration of Tollen’s reagent and reaction time are 2.4 mM of Ag^+^ and 90 s separately.

In addition to the abovementioned optimization of the MRS sensor, the coupling ratio of CAT and Ab_2_ on the surface of the PS particles and the concentration of H_2_O_2_ should also be considered in the process of detecting AFP.

The coupling ratio is a critical factor for the recognition reaction and the effect of signal amplification. When the molar ratio of CAT to Ab is 10:1, ΔT_2_ reaches the largest value (Supplementary Figure S8A); thus, we select 10:1 as the optimized molar ratio of CAT/Ab_2_ for the next steps.

We next explore the effect of H_2_O_2_ concentration on the detection performance, since CAT could degrade H_2_O_2_ to affect the formation of Ag-MNPs_30_ and result in the change of ΔT_2_. Although the ΔT_2_ value shows best performance at 500 µM of H_2_O_2_, a higher concentration leads to a narrower detection range for detecting AFP. In consideration of both sensitivity and detection range, we choose 250 µM H_2_O_2_ for further experiments (Supplementary Figure S8B).

### Sensitivity and Selectivity

**FIGURE 6:**
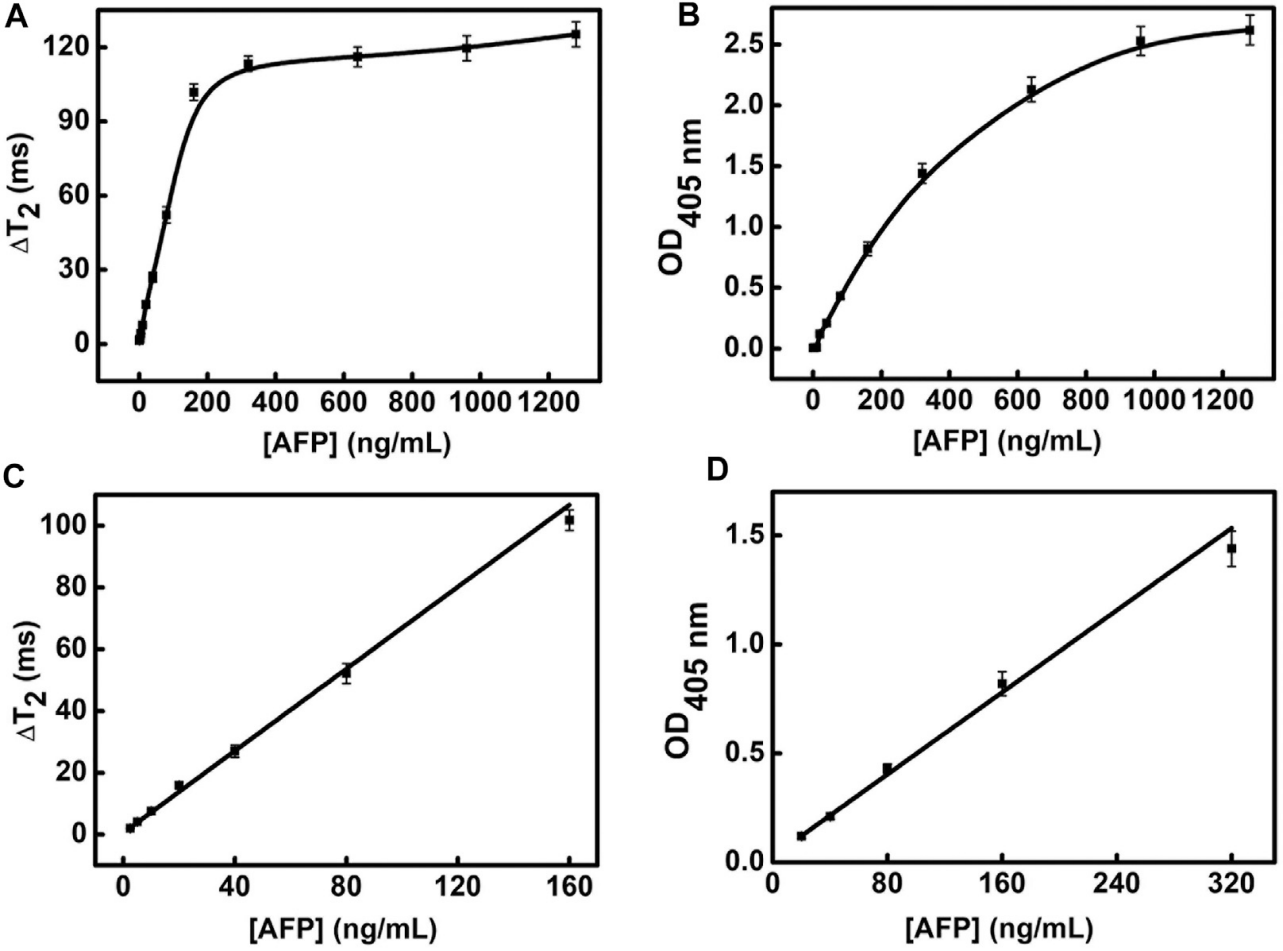
The sensitivity of MRS sensor and pNPP-based ELISA for detecting AFP. **(A)** The detection range of MRS sensor for detecting AFP. **(B)** The detection range of pNPP-based ELISA for detecting AFP. **(C)** The linear range of MRS sensor for detecting AFP. **(D)** The linear range of pNPP-based ELISA for detecting AFP.

Under the above optimized conditions, we compare the analytical performance of the MRS sensor and conventional pNPP (para-nitrophenylphosphate)-based ELISA for the detection of AFP. AFP is a tumor marker for the diagnosis of primary liver cancer (Jalanko et al., 1978; Shen et al., 2020). A high AFP generally means the occurrence of liver cancer. The content of AFP in normal human serum is less than 20 ng/ml. In the AFP detection based on MRS sensor, we employ the MB_S_ with Ab_1_ and PS microparticles coupled with Ab_2_ and CAT as immunological carriers to accomplish mixture, enrichment, and separation. In the redox system with H_2_O_2_ as the reducing agent, the concentration of AFP depends on the ΔT_2_ signal caused by the degree of Ag-MNPs_30_ aggregation. The ΔT_2_ increases as the concentration of AFP changes from 0 to 1,280 ng/ml, and there is a linear relationship from 2.5 to 160 ng/ml. The linear equation is $y\;=\;0.646x+1.019\;\left(R^{2}=0.97\right)$, and the LOD of MRS sensor for detecting AFP is 0.56 ng/ml (Figures 6A,C). In comparison, the linear range of the pNPP-based ELISA for the detection of AFP is 20–320 ng/ml. The linear equation is $y\;=0.00471x+0.01217\;\left(R^{2}=0.99\right)$, and the LOD is 7 ng/ml (Figures 6B,D). Thus, the sensitivity of MRS sensor for AFP detection has been improved about 12.5 folds compared with the conventional pNPP-based ELISA.

We also investigated the selectivity of the MRS sensor for detecting AFP (160 ng/ml), by using CEA (100 ng/ml) and human IgG (5 mg/ml) as the interferent (Supplementary Figure S9). We chose CEA as the interferent because CEA is also one of the liver cancer markers in human serum. We chose human IgG as the interferent because human serum contains a large amount of IgG (7–16.6 mg/ml), which may interfere the detection of AFP. The relative errors of ΔT_2_ between AFP with and without interfering agents were 1.6% (CEA as the interferent) and 5.4% (human IgG as the interferent). Moreover, ΔT_2_ of CEA, IgG are far lower than AFP. It suggests that the selectivity of the MRS sensor provides a prerequisite for the practical application for detections of AFP in complex samples.

### Real Sample Analysis

**FIGURE 7:**
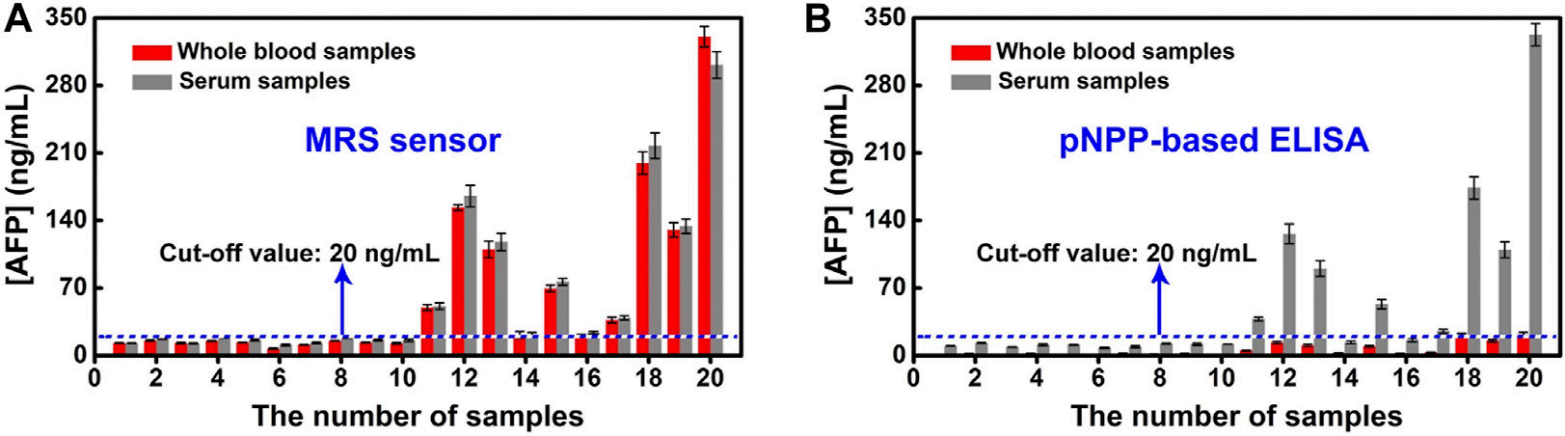
**(A)** Correlation between the detection of AFP in whole blood samples and serum samples by MRS sensor. **(B)** Correlation between the detection of AFP in whole blood samples and serum samples by the pNPP-based ELISA.

To confirm the application of our approach, we compare MRS sensor with pNPP-based ELISA in detecting AFP with 20 whole blood samples and 20 serum samples. The MRS sensor based on magnetic signals can directly detect AFP in both whole blood samples and serum samples (Figure 7A). The OD intensity of pNPP-based ELISA is determined by spectrophotometric colorimetry, which is applicable to serum samples instead of the red whole blood samples in clinical diagnosis (Supplementary Figure 7B). In 20 serum samples, samples from 11 to 20 are all detected to be AFP positive by the MRS sensor, and other samples are detected to be AFP negative. The difference is that samples 14 and 16 appear false negative (Supplementary Figure S10). The quantitative results of the MRS sensor for detecting AFP in serum samples agree well with those of pNPP-based ELISA with a correlation coefficient of 0.97 (Supplementary Figure S11).

To show the benefit of our method, we have compared the analytical performance of the MRS sensor with that of the MRS sensor in other reported works (Fan et al., 2014; Chen et al., 2016) for AFP detection in terms of detection time, LOD, performance of operations, consumption of reagents, dependence of devices, and sample types (Table 1). As we can see, the MRS sensor we developed shows a relative lower LOD, requires minimal sample, and can detect target in whole blood samples directly, while ELISA and CLIA can only be used in serum. Although GICA has good sensitivity as the MRS sensor, it requires higher consumption of reagents.

**TABLE 1:** The characteristics of the MRS sensor are compared with those of the MRS sensor in other methods.

Detection method	MRS sensor	ELISA	GICA	CLIA
Detection time	25 min	>4.5 h	30 min	40 min
LOD	0.56 ng/ml	2 ng/ml	0.21 ng/ml	1.5 ng/ml
Performance of operations	Simple	Complicated	Simple	Complicated
Consumption of reagents	Low	High	High	High
Dependence of devices	Dependent	Dependent	Independent	Dependent
Sample	Whole blood, serum	Serum	Whole blood, serum	Serum
References	This work	ELISA kit	Chen et al. (2016)	Fan et al. (2014)

## Conclusion

In conclusion, we employed a microfluidic chip–based MRS sensor *via* enzyme-triggered nanoparticle assembly to replace elaborate manual operation and enhance the detecting sensitivity. Compared with conventional pNPP-based ELISA, the MRS sensor we developed is a competitive sensor for detecting whole blood samples because of its low background signal and high sensitivity. This work also has the potential of high throughput detection, and we will focus on optimizing the microfluidic chip, automatic detection equipment, and immune reaction system to achieve the application of multiple marker detection in the clinical diagnosis.

## Data Availability

The original contributions presented in the study are included in the article/Supplementary Material, further inquiries can be directed to the corresponding authors.
