# Supplementary material for: A Microfluidic Chip-Based MRS Immunosensor for Biomarker Detection via Enzyme-Mediated Nanoparticle Assembly
Source: Front Chem. 2021 May 28;9:688442. doi: 10.3389/fchem.2021.688442 (PMC8193930; doi:10.3389/fchem.2021.688442)
Supplement: Supplementary file 1 [file DataSheet1.docx]

Supplementary Material

**A microfluidic chip-based MRS immunosensor for biomarker detection via enzyme-triggered nanoparticles assembly**

Binfeng Yin^1*^, Changcheng Qian^1^, Teng Zhou^3^, Xinhua Wan^1^, Songbai Wang^2*^

^1^School of Mechanical Engineering, Yangzhou University, Yangzhou 225127, China

^2^School of Chemistry and Chemical Engineering, Shanxi University, Taiyuan 030006, China

^3^Mechanical and Electrical Engineering College, Hainan University, Haikou 570228, China

^*^ Corresponding author


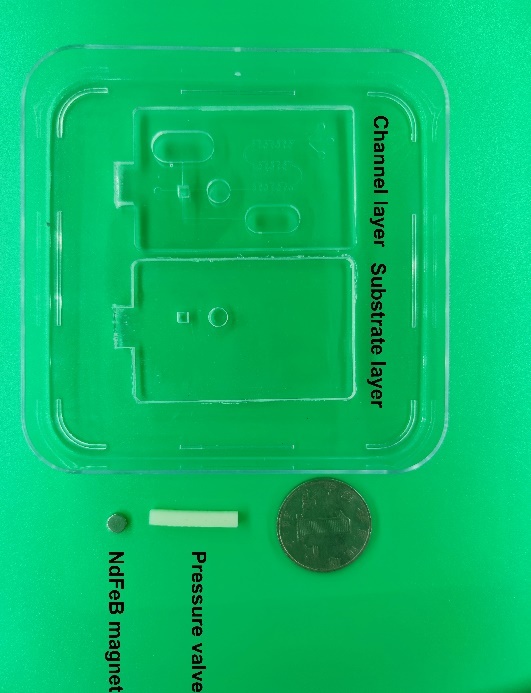


**Figure S1.** The components of SSMC include PDMS channel layer, PDMS substrate layer, NdFeB magnet and 3-holes pressure valve.


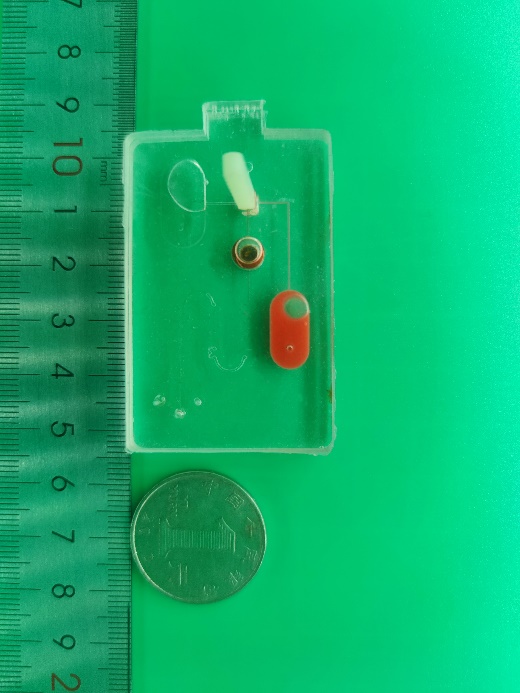


**Figure S2.** Picture of SSMC when detecting whole blood sample.


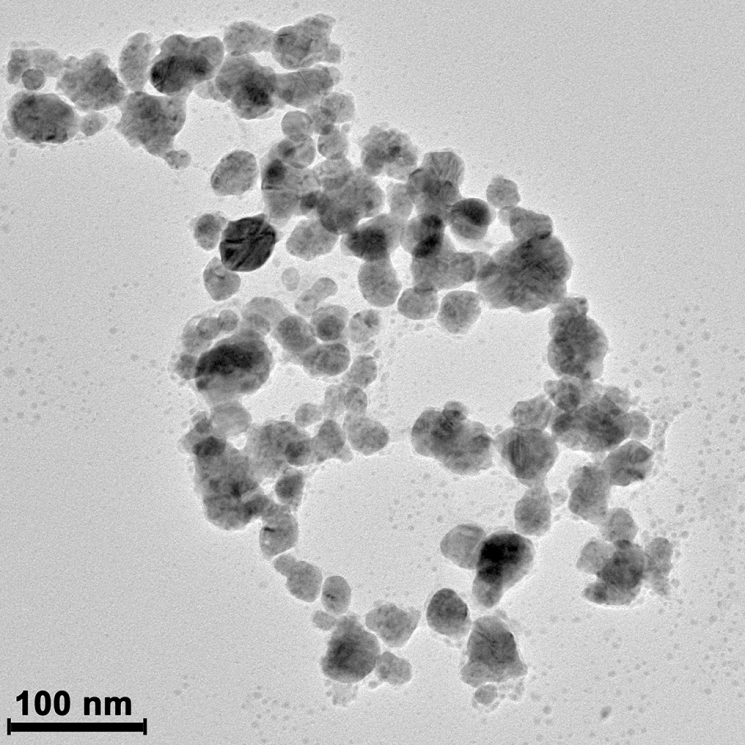


**Figure S3.** (A) The TEM image of Ag NPs.





**Figure S4.** UV−vis spectra of MNPs_30_-COOH, MNP_30_-NH_2_, Ag NPs, and Ag-MNPs_30_-COOH and Ag-MNPs_30_-NH_2_.





**Figure S5.** The T_2_ value for detecting MNPs_30_ at different concentrations from 0 to 50 μg/mL.


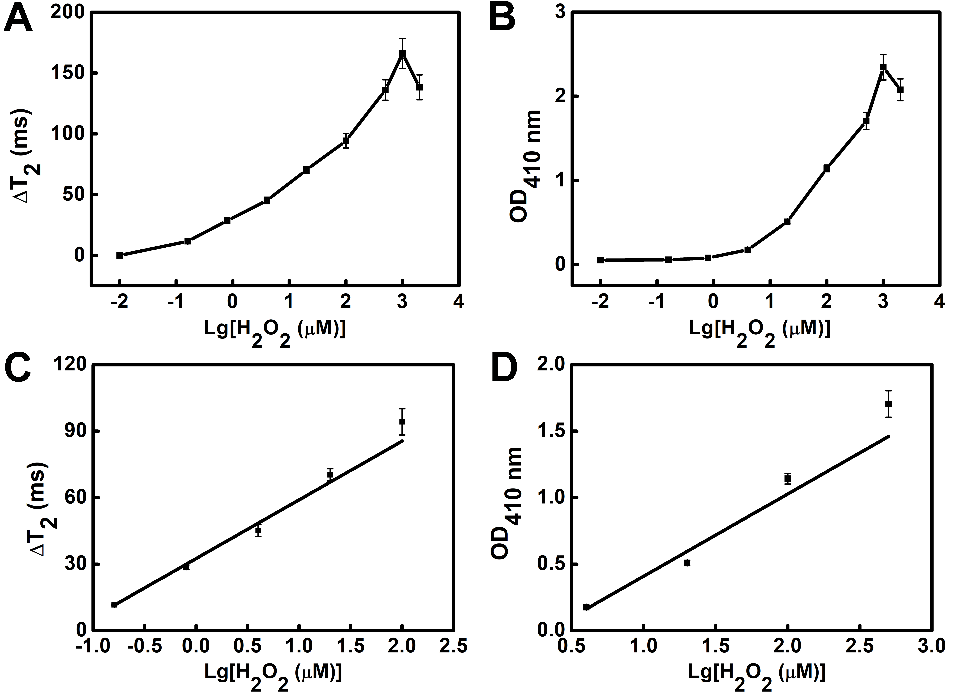


**Figure S6.** The comparison of the MRS sensor (A, C) and optical readout (B, D) for detection of H_2_O_2_.


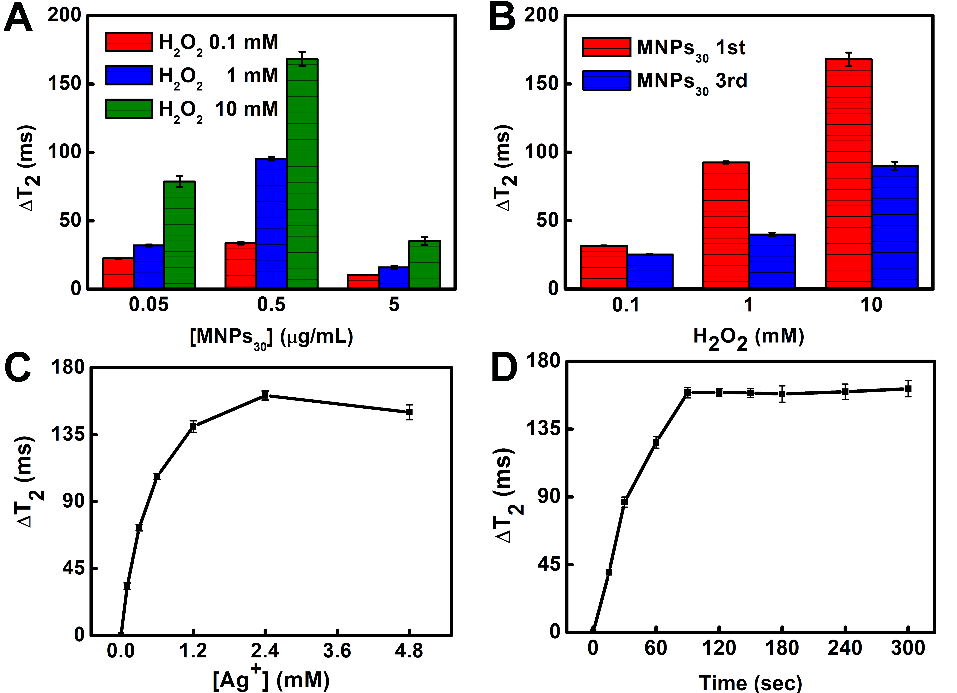


**Figure S7.** Optimization conditions for detection of H_2_O_2._ (A) MNPs_30_ at different concentrations from 0.05 to 5 μg/mL. (B) Different sequence of adding MNPs_30_. (C) The effect of Ag^+^ concentration. (D) The effect of reaction time.


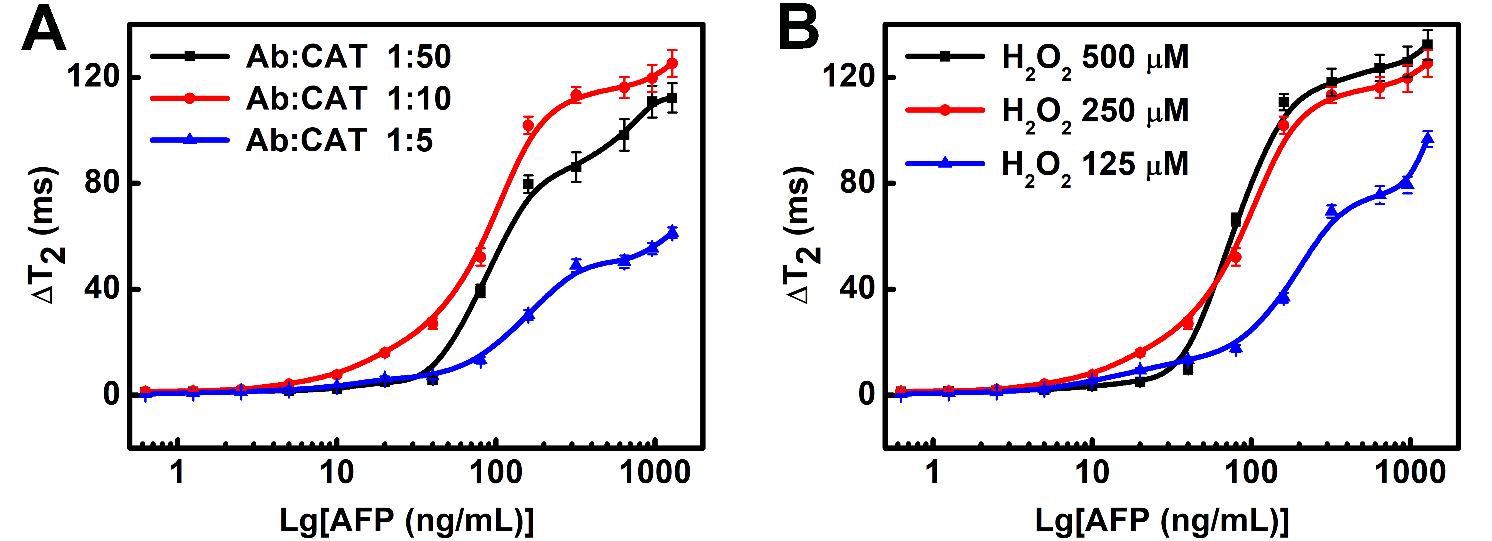


**Figure S8.** Optimization conditions for detection of AFP. (A) The effect of molar ratio of Ab/CAT. (B) The effect of H2O2 concentration.





**Figure S9.** The selectivity of MRS sensor for detection of AFP.





**Figure S10.** The comparison of MRS sensor and pNPP-based ELISA in serum sample.





**Figure S11.** The correlation coefficient of the MRS sensor and pNPP-based ELISA for AFP detection.
